# Pluripotent and Multipotent Stem Cells Display Distinct Hypoxic miRNA Expression Profiles

**DOI:** 10.1371/journal.pone.0164976

**Published:** 2016-10-26

**Authors:** Rahul Agrawal, Tina P. Dale, Mohammed A. Al-Zubaidi, Prit Benny Malgulwar, Nicholas R. Forsyth, Ritu Kulshreshtha

**Affiliations:** 1 Department of Biochemical Engineering and Biotechnology, Indian Institute of Technology, Delhi, India-110016; 2 Guy Hilton Research Centre, Institute of Science and Technology in Medicine, University of Keele, Thornburrow Drive, Hartshill, Stoke-on-Trent, Staffordshire, ST4 7QB, United Kingdom; 3 College of Pharmacy, Al-Mustansiriyah University, Baghdad, Iraq; 4 Department of Pathology, All India Institute of Medical Sciences, New Delhi, India-110029; University of Wisconsin-Madison, UNITED STATES

## Abstract

MicroRNAs are reported to have a crucial role in the regulation of self-renewal and differentiation of stem cells. Hypoxia has been identified as a key biophysical element of the stem cell culture milieu however, the link between hypoxia and miRNA expression in stem cells remains poorly understood. We therefore explored miRNA expression in hypoxic human embryonic and mesenchymal stem cells (hESCs and hMSCs). A total of 50 and 76 miRNAs were differentially regulated by hypoxia (2% O_2_) in hESCs and hMSCs, respectively, with a negligible overlap of only three miRNAs. We found coordinate regulation of precursor and mature miRNAs under hypoxia suggesting their regulation mainly at transcriptional level. Hypoxia response elements were located upstream of 97% of upregulated hypoxia regulated miRNAs (HRMs) suggesting hypoxia-inducible-factor (HIF) driven transcription. HIF binding to the candidate cis-elements of specific miRNAs under hypoxia was confirmed by Chromatin immunoprecipitation coupled with qPCR. Role analysis of a subset of upregulated HRMs identified linkage to reported inhibition of differentiation while a downregulated subset of HRMs had a putative role in the promotion of differentiation. MiRNA-target prediction correlation with published hypoxic hESC and hMSC gene expression profiles revealed HRM target genes enriched in the cytokine:cytokine receptor, HIF signalling and pathways in cancer. Overall, our study reveals, novel and distinct hypoxia-driven miRNA signatures in hESCs and hMSCs with the potential for application in optimised culture and differentiation models for both therapeutic application and improved understanding of stem cell biology.

## 1. Introduction

Human embryonic and mesenchymal stem cell (hESCs and hMSCs, respectively) precursors are thought to reside in physiologically hypoxic environments [1, 2]. Furthermore the physiological reintroduction of clinically relevant stem cells for therapeutic application, whether intravenously or intra-tissue will inevitably result in either acute or chronic hypoxic exposure to the transplanted materials [3]. *In vitro* experimentation has established that hypoxic culture of hESC correlates closely with increased clonogenicity, reduced spontaneous differentiation, increased genetic stability, and transcriptional homogeneity alongside improved epigenetic profiles [4, 5, 6, 7]. Coupled to this it is also reported that the utilisation of hypoxic culture conditions in the recovery of hMSC from bone marrow, and other tissues e.g. fat, results in dramatic improvements in individual stem cell yield (via Colony-Forming Unit- Fibroblastic quantification), enhanced scale-up, and reduced transcriptional alteration (vs. normoxic cultured (21% O_2_) cells) [8]. Of clinical relevance recent reports have suggested that ex-vivo hypoxic pre-conditioning of MSCs results in enhanced survival post-transplant via resistance to intrinsic and extrinsic death signals [3]. Taken together these investigations suggest that either control of modulation of hypoxic signalling, or hypoxia-regulated genes could be of benefit to the regenerative medicine industry. This identifies a need to identify novel players in hypoxic signalling that may serve as candidates for the enhancement of stem cell based therapies.

MicroRNAs (miRNAs), a class of small non-coding RNAs, have emerged as key players in cellular transformation and development [9]. A role for miRNAs in governing aspects of stem cell biology as biological switches for self-renewal, fate acquisition, and differentiation has emerged [10]. In addition to the roles detailed above it has become clear that miRNAs act as critical mediators of hypoxia signalling [11]. Specifically, hypoxia regulated miRNAs (HRMs) have been demonstrated to have roles in cell cycle modulation, apoptosis, DNA repair pathways, angiogenesis, metabolism, metastasis, proliferation and resistance to anticancer therapy [12–15]. To date a single report details hypoxia-driven modulation of miRNA expression in murine MSCs where MiR-210, -23a and miR-21 promoted the survival of MSCs exposed to hypoxia [16].

Surprisingly, there are as yet no reports detailing hypoxia-driven modulation of miRNA expression in human stem cells; embryonic or adult. We therefore sought to establish whether both embryonic (hESC) and adult (hMSC) displayed a common HRM profile in response to a hypoxic culture setting. We have identified highly divergent HRM signatures in hESCs and hMSCs. Importantly, target genes of these HRMs were linked to key regulatory pathways with roles in stem cell fate determination. This work opens up several avenues for further investigation in the field of hypoxic stem cell biology. Identification of HRMs that improve stem cell survival may have strong beneficial implications for regenerative and transplantation medicine.

## 2. Materials and Methods

### 2.1. Cell culture

This study doesn't require any ethical statement. The hMSCs were isolated from commercially sourced bone marrow (Lonza) which does not require ethics and the hESCs were used under approval from the UK Stem Cell Bank (UKSCB). HESCs were cultured either as described previously (SHEF1) [17] or in defined conditions (SHEF2). SHEF1 were cultured in a mouse embryonic fibroblast (MEF)-conditioned Knockout-DMEM supplemented with 20% Knockout-Serum Replacement, 1 mM L-glutamine, 1% non-essential amino acids, 100 mM β-mercaptoethanol (all Life Technologies, Paisley, UK), and 4 ng/mL bFGF (Sigma, Poole, UK). Cells were expanded on flasks coated with a Matrigel (Becton Dickinson, Bedford, MA) substrate in preconditioned media. Prior to use, conditioned media was further supplemented with an additional 4 ng/mL bFGF. Cells were passaged enzymatically at 70–80% confluency using Trypsin/EDTA and media changed daily. SHEF2 were cultured in Essential 8 defined medium on vitronectin-coated tissue culture plates. At 70–80% confluency, cells were passaged with 0.5 mM EDTA treatment; following passage media was changed at 48 hours and daily thereafter. All reagents from Life Technologies (Paisley, UK) unless otherwise stated. Cells were maintained in standard incubators at 37°C in a humidified 5% CO_2_ atmosphere. For maintenance of hypoxic conditions, cells were grown continuously for ≥10 passages or approximately 6 weeks in a hypoxia workstation (*SCI-tive*, Ruskinn, UK) at 2% O_2_, 5% CO_2_, 37°C. Cells were used at passages 61–63 for RNA extraction.

Human MSCs were isolated from commercially sourced bone marrow aspirate (Lonza, USA) retrieved from the iliac crest following detailed methodology published in Kay *et al* 2015 [8]. Briefly, whole bone marrow was seeded at a density of 10^5^ mononuclear cells (MNC)/cm^2^ on 10 ng/mL fibronectin-coated flasks in each condition (21% O_2_ and 2% O_2_). MNC were seeded into DMEM (4.5g/L) 5% FBS, 100 U/mL penicillin, 100 U/mL streptomycin, 1% NEAA, and 1% L-Glutamine (all Lonza, UK). Penicillin and streptomycin were included initially to counteract the inherent infection risk associated with the culture of primary materials. After 7 days half of the culture medium was replaced with fresh antibiotic-free media; after 14 days media was replaced completely again without the inclusion of antibiotic. At day 21 the cells were harvested at passage 0 for RNA isolation. For maintenance of hypoxic conditions, cells were recovered and cultured continuously in a hypoxia workstation (*SCI-tive*, Ruskinn, UK) at 2% O_2_, 5% CO_2_, 37°C prior to RNA extraction.

### 2.2. Cell lysis and homogenisation

In brief, cell monolayers were washed with PBS and lysed using the RLT lysis buffer from the RNeasy minikit (Qiagen, UK). A cell scraper was used to detach cells from the culture flask and the resulting lysate transferred to a QIAshredder spin column (Qiagen) for homogenisation. The QIAshredder column was centrifuged for 2 minutes at 13200 RPM, the column removed and the lysate stored at -80°C until required.

### 2.3. RNA extraction

RNA was extracted from cell lysates according to manufacturer’s instructions using the RNeasy Minikit. The concentration and purity of RNA extracted was measured using a NANODROP 2000c spectrophotometer (Thermo Scientific) and RNA was subsequently stored at -80°C.

### 2.4. Microarray expression profiling and analysis

MiRNA expression data was generated from the Affymetrix GeneChip^®^ miRNA arrays via iLife Discoveries. Normalization was performed with Expression Command Console and subsequent analysis with GeneSpring GX 11.5 Software. A ± 2 fold change cut off was used for identifying differentially expressed miRNAs (S1 Table).

### 2.5. Prediction of HREs in the promoter of miRNAs

HRE detection was performed within the promoter region (5KB upstream of the 5’end of the pre-miRNA) of candidate miRNAs. The upstream region was extracted from Ensembl genome browser. HRE consensus sequences are as described elsewhere [11]. The following consensus sequences were used to search for HREs: Q3-GNNKACGTGCGGNN, Q5-NGTACGTGCNGB, Q6- NRCGTGNGN, (N—Adenine / Guanine / Cytosine / Thymine, B—Guanine / Thymine / Cytosine, R—Guanine / Adenine) using python 3.2 software. HIF1A binding sites were identified within promoter regions using the PROMO prediction program [18, 19].

### 2.6. Chromatin Immunoprecipitation-qPCR (ChIP-qPCR)

The Low Cell ChIP Kit (M/S Diagenode, Belgium) protocol was followed with some minor modifications. Briefly, cells were cross-linked using 1% formaldehyde in PBS with the reaction stopped after 10 min using 100 μl of 125 mM glycine. The cells were then washed with PBS followed by suspension in ChIP buffer. The chromatin was next fragmented using Diagenode Bioruptor plus. Fragmented chromatin (equivalent to 1 million cells) was immunoprecipitated using HIF-1 antibody (M/s Santa Cruz) and IgG antibody (M/s Abcam, USA) as per Low Cell ChIP kit guidelines. After overnight incubation, the beads were washed and immunoprecipitated and input DNAs were proceeded for DNA isolation using IPure kit (M/s Diagenode, Belgium) following the manufacturer’s instructions. qPCR reactions were performed using 3μl of DNA in a CFX96^™^ Real time system (BIO-RAD) using SyBr green dye with primers encompassing HREs in promoter sequence of various miRs and ACTB (negative control). Enrichment was expressed as the percent input by using the following formula: Percentage of total input = 100×2^[Ct (ChIP)—(Ct input—log2 (input dilution factor)]

### 2.7. Target prediction and pathway analysis

Probable targets for differentially regulated miRNAs were identified with the DIANA-MicroT-CDS prediction program [20]. DIANA-mirPath was used for pathway analysis of differentially expressed microRNAs in response to hypoxia [21]. A list of probable targets was determined via the inverse correlation of expression with the differentially expressed miRNA matched to previously published gene expression data of hypoxic cultured hESC and hMSCs under identical conditions [22, 8].

### 2.8. MiRNA quantitation

Candidate miRNAs were reverse transcribed to cDNA using specific stem-loop RT primers (S2 Table). Quantitation was performed on a CFX96^™^ real time system (Bio-RAD) using cDNA specific forward primer and a universal reverse primer as listed in S2 Table. RNU6B was used for normalization in all the samples. A list of primers and sequences is given in S2 Table.

## 3. Results

### 3.1. MicroRNA signature of hypoxia in human embryonic and mesenchymal stem cells

Previous studies, including ours, have described hypoxia-induced differential gene expression in hESCs and hMSCs [22, 23, 8], but hypoxia-driven miRNA regulation in hESCs and hMSCs remains undescribed thus far. SHEF1 (hESCs) cells and hMSCs were exposed continuously to normoxia (21% O_2_) or hypoxia (2% O_2_) followed by RNA extraction and determination of miRNA expression using Affymetrix GeneChip^®^ miRNA Arrays.

**Fig 1:**
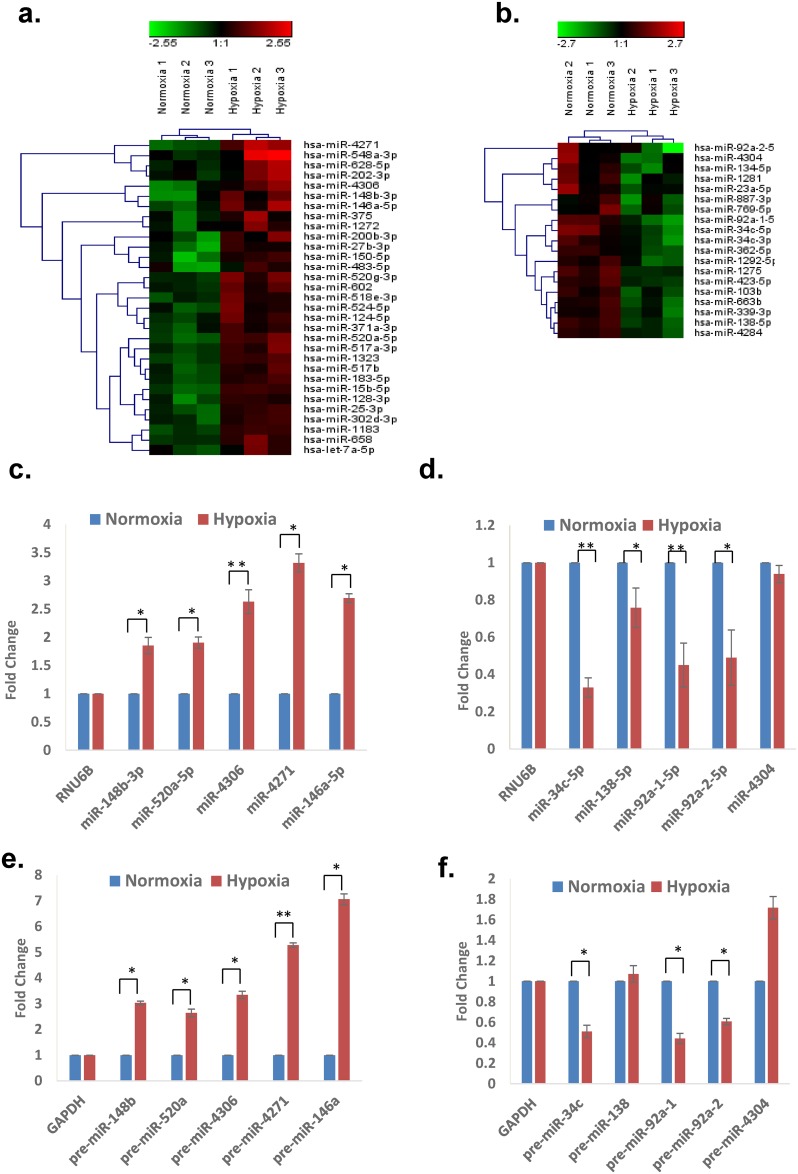
The miRNA signature of hypoxia in hESCs. Hierarchical clustering of hypoxia **(a)** induced and **(b)** down-regulated miRNAs (> 2 fold, p<0.05) in response to hypoxia (2% O_2_) in SHEF1 hESC. Quantitative RT-PCR data of upregulated **(c)** mature miRNAs & **(e)** pre-miRNAs and downregulated **(d)** mature miRNAs & **(f)** pre-miRs of HRMs in SHEF1 cell line. Graphical data points in c, d, e and f represent mean ± S.D. of a minimum of three independent experiments. (*P>0.01 and <0.05, **P<0.01).

Comparison of normalized normoxic and hypoxic hESCs miRNA profiles identified differential expression of 50 miRNAs (>2 fold difference, p<0.05); 31 up-regulated and 19 down-regulated in response to hypoxia (Fig 1a and 1b, S1 Table). Upregulation (miR-4271,-4306, -520a-5p, -148b-3p and miR-146a-5p) and down regulation (miR-92a-1-5p, -92a-2-5p,-34c-5p, -138-5p and miR-4304) were checked in the array-based line (SHEF1) and in a second independent line (SHEF2) using quantitative stem loop RT-PCR (Fig 1c and 1d, S1a and S1b Fig). However, while stem loop qRT-PCR results confirmed the down-regulation of miR-92a-1-5p, miR-92a-2-5p and miR-34c-5p, the level of miR-4304 remained largely unaffected (Fig 1d, S1b Fig).

**Fig 2:**
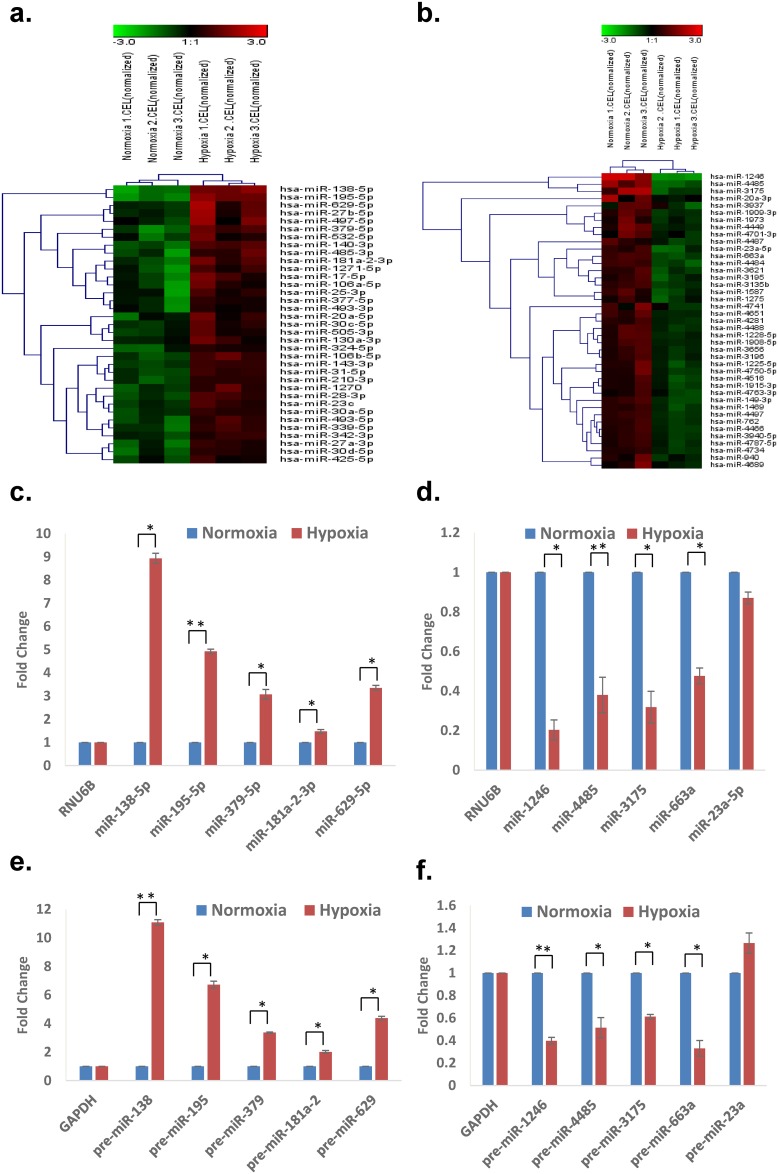
The miRNA signature of hypoxia in hMSCs. Hierarchical clustering of hypoxia **(a)** induced and **(b)** down-regulated miRNAs (> 2 fold, p<0.05) in response to hypoxia (2% O_2_) in human bone marrow derived MSCs. Quantitative RT-PCR data of upregulated **(c)** mature miRNAs and **(e)** pre-miRNAs and downregulated **(d)** mature miRNAs and **(f)** pre-miRs of HRMs in human bone-marrow derived MSCs. The graphical data points in c, d, e and f represent mean ± S.D. of at least three independent experiments. (*P>0.01 and <0.05, **P<0.01).

To explore uniformity of hypoxic miRNA expression modulation in stem cell populations we next explored hMSC isolated from bone marrow. We identified 76 differentially expressed miRNAs in hMSC (35 up-regulated and 41 down-regulated) in response to hypoxia (Fig 2a and 2b, S1 Table). Up-regulated (miR-138-5p, -195-5p, -379-5p, -181a-2-3p, and miR-629-5p) and down-regulated (miR-1246, -4485, -3175, and miR-663a) miRNA expression was confirmed with quantitative stem loop RT-PCR in hMSC derived RNA from two independent donor bone marrow samples (Fig 2c and 2d, S2a and S2b Fig). We next checked whether HRMs are affected by hypoxia at the transcriptional level or due to aberrations in miRNA processing at post-transcriptional level. For this, we first checked the levels of miRNA processing factors—Drosha and DICER under normoxia and hypoxia. We found that levels of both miRNA biogenesis factors are downregulated under hypoxia in hESCs and hMSCs (S1c and S2c Figs). We then checked the levels of corresponding pre-miRNAs under normoxia and hypoxia and found their regulation was consistent with their mature counterparts in both stem cell types suggesting that the hypoxic induction of these miRNAs was mainly at the transcriptional level (Figs 1e and 1f and 2e and 2f).

Taken together the hypoxic miRNA profiles of hESCs and hMSCs were largely distinct with only miR-25-3p (upregulated), mir-1275 and miR-23a-5p (both downregulated) overlapping between the two stem cell populations. Further evidence of distinct behaviour was noted in relation to miR-138-5p which was downregulated and upregulated by hypoxia in hESC and hMSC, respectively.

### 3.2. Hypoxic regulation of miRNA clusters in hESCs and hMSCs

**Fig 3:**
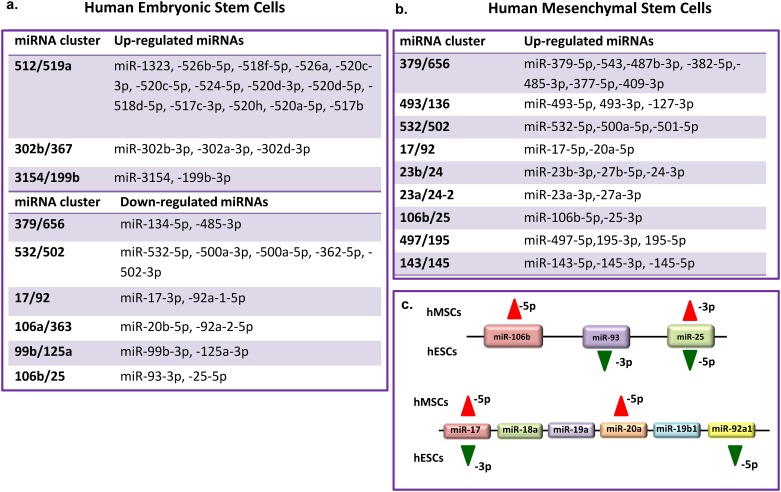
Co-regulation of miRNA clusters by hypoxia. Tables representing hypoxia up-regulated and down-regulated miRNA clusters in **(a)** SHEF1 cells, **(b)** bone marrow derived MSCs, and **(c)** preferential induction of the distinct members of the same cluster by hypoxia.

We next sought to determine co-regulation of miRNA clusters by hypoxia. To determine the overall trend of differential expression within the miRNA cluster, we considered all miRNAs showing >±1.5 fold change. We identified 3 miRNA clusters which contained 19 up-regulated miRNAs while a further 6 miRNA clusters contained 15 down-regulated miRNAs in hESCs (Fig 3a). The miR-512/519a cluster was highly represented containing 14 upregulated miRNA while 2 members of the miR-17/92 cluster and its paralogs miR-106a/363 and miR-106b/25 showed down-regulation in hypoxic hESCs. In the same manner, 25 up-regulated miRNAs lay within 9 miRNA clusters in hMSCs while 7 members of the miR-379/656cluster and 3 members of the miR-532/502 cluster showed up regulation (Fig 3b).

Further evidence of the distinct hESC and hMSC HRM profiles emerged via miRNA clusters- miR-379/656, mir-532/502, miR-17/92 and its paralog miR-106b/25 being downregulated by hypoxia in hESCs while conversely up-regulated in hMSCs. For instance miR-17/92 cluster members were upregulated in hMSC (miR-106b-5p and miR-25-3p) but downregulated (miR-93-3p and miR-25-5p) in hESCs (Fig 3c). This suggests preferential induction of distinct members of the same cluster by hypoxia in developmentally and potency distinct stem cell types.

### 3.3. Hypoxia inducible factors (HIFs) and putative roles in miRNA induction

**Fig 4:**
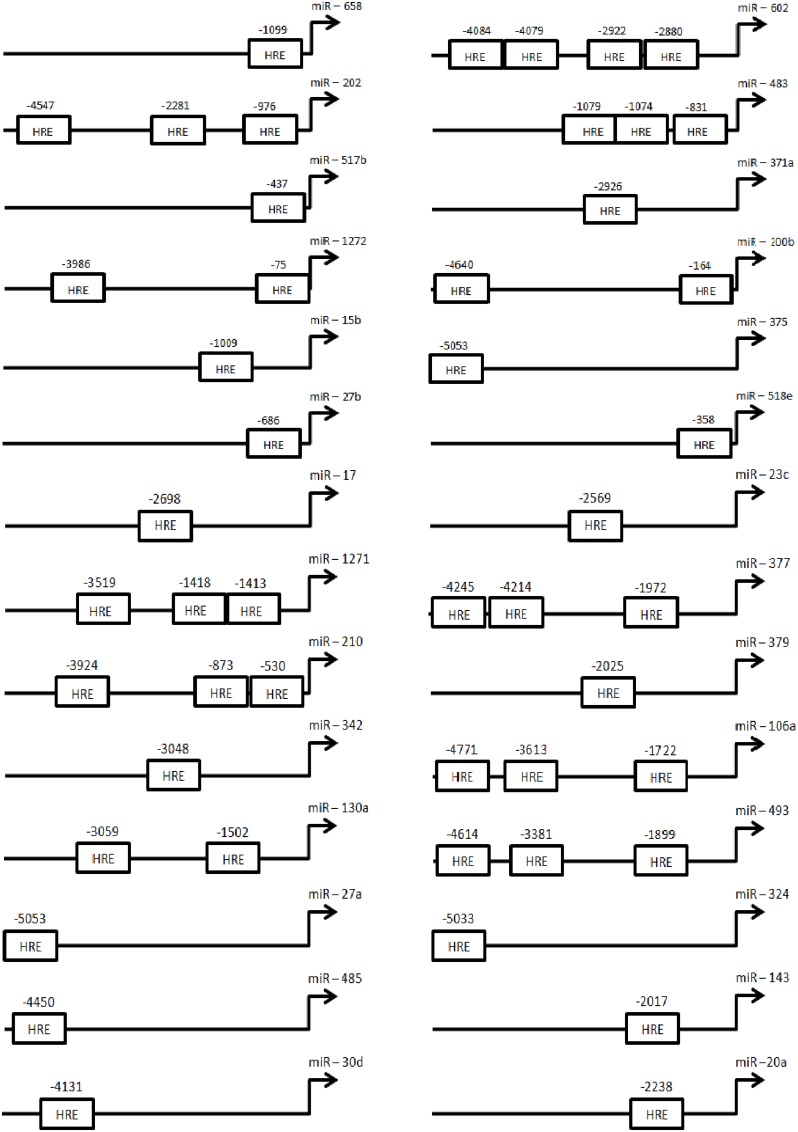
HREs location in HRM promoters. The 5kb upstream region of hypoxia induced miRNAs was screened for the presence of HREs in their promoter using PROMO prediction program. Figure showing relative positions of predicted HREs in the promoter region.

Many hypoxia regulated transcripts contain consensus HIF responsive elements (HRE) in their promoter regions which promote HIF1/HIF2 binding and induction of transcription [11]. The 5 kb region upstream of HRMs was therefore examined for the presence/absence of the HRE consensus sequence. Of the identified HRM’s 97% of upregulated species contained either Q3 or Q5 consensus sequences at >0.75 (S3 Table). Further analysis revealed HIF1A binding site consensus sequences in 12 and 17 upregulated miRNAs for hESC and hMSC, respectively, where strong scores (≤0.85) suggested HIF1A regulation (Fig 4).

**Fig 5:**
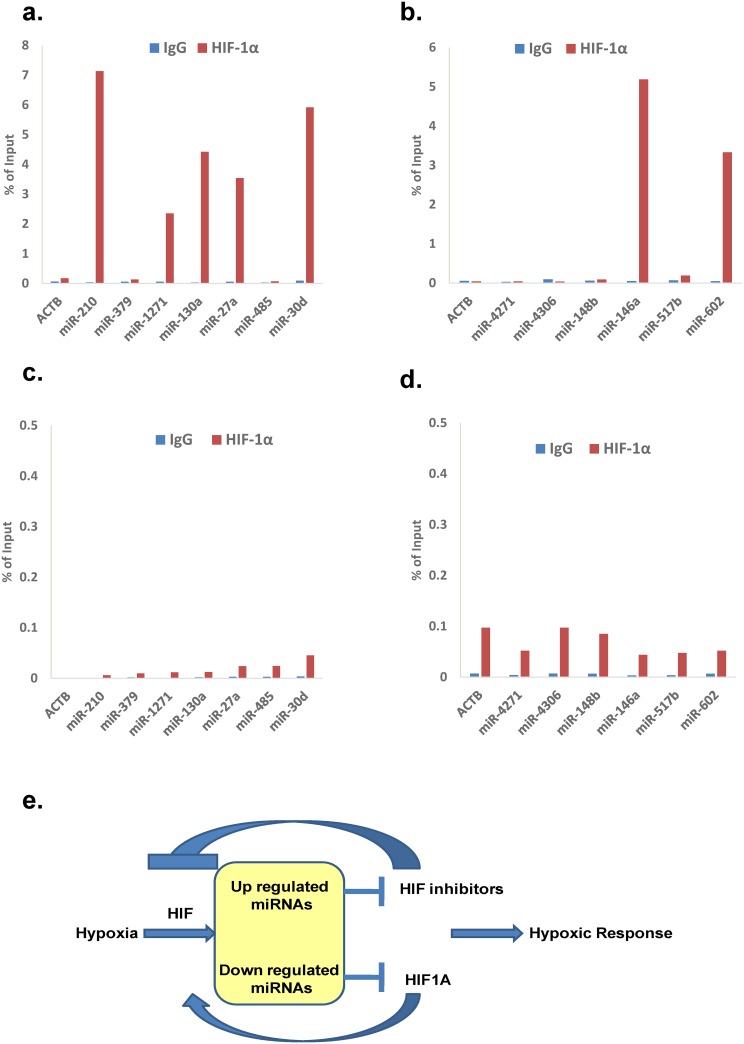
Transcriptional regulation of HRMs. ChIP assay was performed using antibodies against HIF-α or IgG to confirm the binding of HIF-1 α on the putative HREs in the promoters of specific HRMs. qPCR data showing fold enrichment of bound chromatin compared with input in hMSCs grown in **(a)** hypoxic or **(c)** normoxic conditions and hESCs in **(b)** hypoxic and **(d)** normoxic conditions. **(e)** Putative hypoxia signal transduction feedback loop in hESC and hMSCs.

HIF binding to candidate HREs was examined by CHIP-qPCR in hESCs and hMSCs under normoxia and hypoxia. In hypoxia treated hMSCs, direct HIF binding to the cis-elements was observed for 5/7 miRNAs tested (while in hypoxic hESCs the HREs present upstream of miR-146a and miR-602 were found to be bound by HIF-1 (Fig 5a and 5b). HIF binding to the promoter elements of candidate miRNAs was inhibited in normoxic conditions in both types of stem cells (Fig 5c and 5d). This suggests that the induction of specific miRNAs under hypoxia is HIF mediated in hESCs and hMSCs.

The presence of HRE and HIF1A-specific binding site consensus sequences led us to hypothesize on the existence of a feedback loop in hypoxia signal transduction in hESCs and hMSCs. We therefore next sought to identify which of our HRMs was predicted to target HIF directly or via targeting genes that regulate HIF levels. The hESC (miR-520a-5p, -4271, and miR-4306) and hMSC (miR-138-5p, -140-3p, -210 and miR-1271) upregulated HRMs were predicted to target HIF pathway inhibitors; HIF1A inhibitor (HIF1AN) and HIF3A, respectively, creating a prospective HIF pathway positive feedback loop. In contrast, downregulated HRMs in hESCs (miR-92a-1-5p and miR-92a-2-5p) were predicted to target HIF1A directly creating a negative feedback loop to suppress HIF signal transduction. Overall, this suggests that specific HRMs may operate as a check and balance system to regulate HIF levels during exposure to hypoxia (Fig 5e).

### 3.4. MiRNA: gene networks

**Fig 6:**
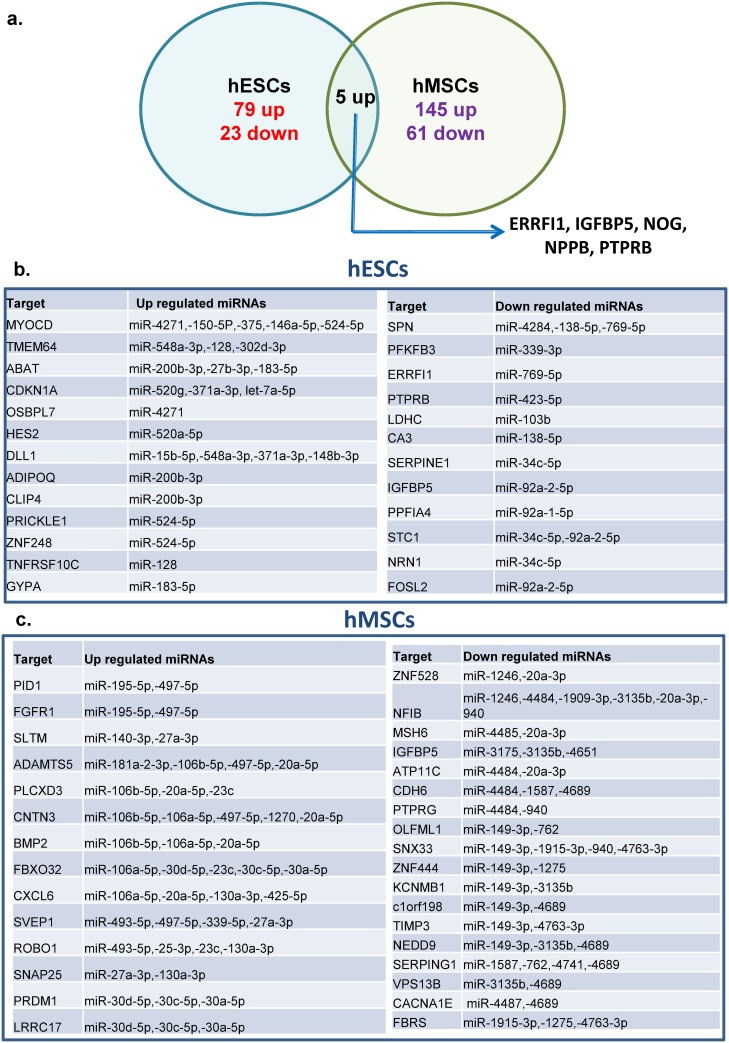
Target identification of hypoxia regulated miRNAs. **(a)** Venn diagram of up and down-regulated genes in response to hypoxia in hESC and hMSCs. **(b, c)** Putative targets HRMs based on the inverse correlation of their expression with previously published hypoxia-induced differential gene expression of hESCs and MSCs, respectively, under identical conditions.

Utilising previously generated transcript array data sets for both hESC and hMSC in normoxia/hypoxia we next sought to determine miRNA:gene correlations by identifying the inverse correlation of expression in miRNA:target pairs [22, 8]. Target identification via DIANA-miRPath analysis revealed 107 and 211 differentially expressed genes in hESC and hMSC, respectively, of which five were commonly up-regulated (ERBB receptor feedback inhibitor 1 or ERRFI1, Insulin-like growth factor binding protein 5 or IGFBP5, Noggin or NOG, Natriuretic peptide B or NPPB and Protein tyrosine phosphatase receptor type B or PTPRB (Fig 6a). Of these only IGFBP5 revealed co-association with downregulated miR-92a-2-5p (in hESC) and downregulated miR-3175,-3135b,-4651 (in hMSC). However, we noted substantial overlap of predicted target transcripts of our HRMs and the hypoxia gene expression profiles (Fig 6b and 6c). We next measured the expression levels of the target transcripts identified in Fig 6b and 6c by qPCR to determine their inverse correlation with HRMs, in both culture settings. Notably, most of the tested genes showed a good inverse correlation with their corresponding miRNAs (S3 Fig).

### 3.5. Pathway analyses

**Fig 7:**
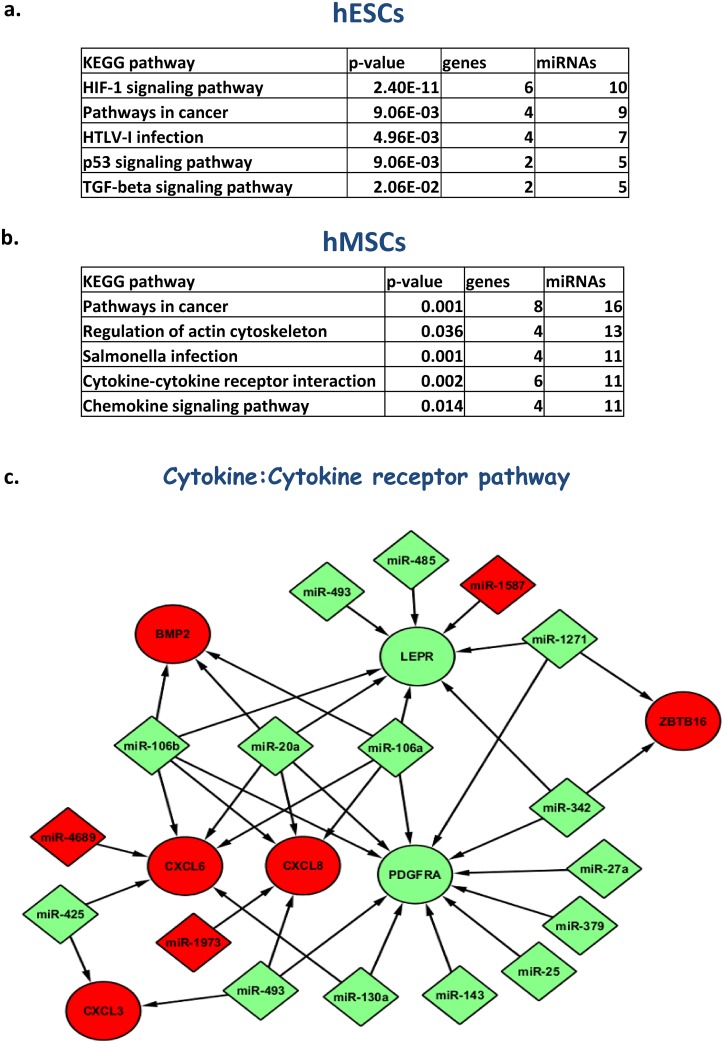
Pathway analysis of hypoxia regulated miRNAs. Pathway association of up and down-regulated HRM in hESC and hMSCs, **(a, b)** respectively. A figure showing miRNA:target gene interaction network for cytokine:cytokine receptor pathway of MSCs drawn using Cytoscape software **(c).** The green colour indicates up-regulation whereas the red colour refers to down-regulation.

Finally we explored pathways enriched by the differentially expressed miRNA target genes in both hESCs and hMSCs. This identified strong associations with the HIF-1 signalling pathway (p = 2.40E-11) for hESC, pathways in cancer (p≤0.000975) for hESC and hMSC, and cytokine-cytokine receptor interaction pathway (p = 0.001933) for hMSCs (Fig 7a and 7b and S4 and S5 Tables). The miRNA:target gene interaction network for cytokine-cytokine receptor pathway is shown in Fig 7c.

## 4. Discussion

In the 15 years since the first demonstration of hypoxic regulation of miRNA expression in a range of cancer cell lines, the hypoxia: miRNA correlation has become widely accepted. Several HRMs have now been shown to be critical regulators of hypoxic signal transduction by regulating cellular properties such as apoptosis, proliferation, metabolism, angiogenesis, DNA repair and stemness [12–15]. While some HRMs have emerged as promising prognostic markers, others are under pre-clinical trials as novel targets for cancer therapy [24, 25]. Surprisingly the link between hypoxia and miRNA expression has not been robustly shown thus far in human stem cells. Our study identifies for the first time the hypoxic miRNA signature in two distinct human stem cell types–hESCs and hMSCs and describes divergence of HRM regulation therein.

A number of miRNA species have been identified in previous studies as having roles in determining hESC biology. These include miR-145 (pluripotency), -148b, -146a (differentiation), -302 (BMP signalling and DNA repair), -195 (apoptosis), -372 (cell division) and miR-9 (migration) [26–31]. Couple to this a number of publications have characterised the range of miRNAs expressed by hESC under standard culture conditions and in agreement with those we noted that three of the top ten upregulated miRNAs in hESCs (miR-4271,-4306, and miR-148b-3p), and members of the miR-512/519a and miR-302b/367 cluster are highly expressed in undifferentiated hESCs [32,33]. Within the miRNAs upregulated by hypoxia in hESC a number have had hypothetical or evidenced targets identified thus far. These include NOG (miR-148b-3p), Jumonji domain containing 3 or JMJD3 (miR-146a-5p), Myocardin or MYOCD (miR-4271, -150-5p, -146a-5p, -524-5p and miR-375) and Transmembrane protein 64 or TMEM64 (miR-548a-3p, -128 and miR-302d-3p) [34–37]. A number of downregulated HRMs have also been described elsewhere where their targets frequently have roles in inhibition of differentiation or the differentiation state. These include NANOG (miR-134), Retinoic acid receptor gamma or RARg (miR-34c) [38, 39]. Similarly, in embryoid bodies, hypoxia downregulated miRNA clusters- miR-17/92 or its paralogs miR-106a/363 or miR-106b/25 have been shown to be upregulated [33]. The miR-17-92 cluster specifically has been shown to target the HIF1A transcript and thus inhibit hypoxia signalling [40]. Taken together it is apparent that up-regulated miRNAs in hypoxic hESCs correlate with inhibition of differentiation whereas down-regulated miRNAs act in the converse manner.

Similarly, miRNAs have been shown to play important roles in the regulation of hMSC biology. MiRNAs such as miR-335, -21, -146a-5p, -377, -494, -141, -10a, -138-5p, -140, -17-5p, -143/145, -302 and miR-210 have been identified as pivotal players in governing specific aspects of hMSC biology related to proliferation, migration, differentiation, angiogenesis, aging, and apoptosis [41–52, 16]. A number of these have been identified here as HRMs. This includes miR-138-5p, miR-140, miR-17-5p and members of miR-143/145 cluster as upregulated HRMs which have been described elsewhere as having a role in suppression of hMSCs differentiation [48–51]. Of note are the upregulated HRMs predicted to target Bone morphogenetic protein 2 or BMP2; miR-106b-5p,-106a-5p and miR-20a-5p, whose repression has recently been associated with enhanced hMSC yields in hypoxic conditions and inhibition of chondrogenesis [8, 53]. Other upregulated HRMs and their targets include Sushi, von Willebrand factor type A, EGF and pentraxin domain-containing protein 1 or SVEP1 (miR-493-5p, -497-5p, -339-5p and miR-27a-3p), TGF-β/BMP signalling or Smads (miR-23b/24 cluster-miR-23b, -27b, -24-1)), and PR domain zinc finger protein 1 or PRDM1 (miR-30a-5p, -30c-5p and miR-30d-5p) [54]. Downregulated HRMs were striking in that for the most part they displayed an associated lack of known targets in hMSC biology but miRs- miR-1246, -4484, -1909-3p, -3135b and miR-940 are known to target Nuclear factor 1 B-type or NFIB which plays a role in the promotion of cortical development and neuronal differentiation [55].

The largely distinct HRM profiles observed between hESC and hMSC illustrate the likely existence of divergent hypoxia signalling pathways in these two cell types. This concept is underlined by a comparison of the hypoxia induced transcriptomes in hESC and hMSC which bear little resemblance to each other. Intriguingly we noted that the canonical upregulated HRM, miR-210 though induced in hMSC, in agreement with other studies [16, 56], was not noted in hypoxic hESC. As miR-210 has been found to be hypoxia-inducible in a wide range of cancer cell lines studied its absence from the list of HRMs of hESCs is intriguing [56]. Overall the hESC upregulated HRMs are unique at this time and have not been described as hypoxia regulated thus far. Conversely a number of previously described upregulated HRMs including miR-138, -195, -181a-2-3p, -485-3p, -210, 17-5p, -27a/b were also upregulated in hypoxic hMSC [56, 57, 58]. Overall, and reflective of the divergent HRM profiles, it is likely that miRNA play important roles in governing hypoxic stem cell behaviour.

We also noticed that hypoxia based regulation of most of these HRMs is mainly at transcriptional level. The appearance of HIF1A binding site consensus sequences in the majority of HRM promoter sequences coupled to predicted targets including HIF or HIF regulating genes was suggestive of the existence of a feedback-loop in HIF signalling. The cell type specific upregulated miRs (miR-520a-5p, -4271 and -4306 for hESCs and miR-140-3p, -210, -485 and -1271 for hMSCs) were predicted, or previously experimentally validated, to target HIF pathways inhibitors-HIF1AN and HIF3A while downregulated miRNAs (miR-138-5p, -92a-1-5p and miR-92a-2-5p in hESCs) were predicted to target HIF1A [56, 58]. The presence of an HRM-driven feedback loop in HIF signalling has been suggested elsewhere via miRNAs such as miR-210, -485, -155 and miR-429 [56, 58, 59, 60] but not in relation to stem cell biology. However, we do acknowledge that a role for other transcription factors such as Nuclear factor kappa B or NFĸB, cAMP response element- binding protein or CREB, Activator protein-1 or AP-1, Specificity protein-1/3 or SP-1/3 etc. cannot be dismissed for the HRMs which do not show the consensus HRE sequences in their upstream region [61]. Alternatively, it remains possible that epigenetic regulation of the HRMs is occurring via hypoxic activation of methyl transferases resulting in induction of expression [62]. Coupled to above it was apparent that HRMs in hESCs had a predicted role in HIF signalling via regulation of other hypoxia associated genes (S4 Fig). Several hypoxia-induced genes with roles in HIF signalling such as cyclin dependent kinase inhibitor 1 A or p21 (CDKN1A), Serine protease inhibitor or SERPINE and 6-phosphofructo-2-kinase/fructose-2,6-bisphosphatase 3 or PFKFB3 showed inverse correlation of expression with the HRMs predicted to target them [63, 64, 65].

Two further pathways enriched in gene ontology analyses of the HRM target genes were cytokine: cytokine receptor (hMSCs) and pathways in cancer (hESCs and hMSCs). In confirmation of our findings previous reports have described oxygen-dependent expression of cytokines and linked them with proliferation and differentiation of hMSCs [8, 66]. A role for HRMs in cytokine expression modulation has also been demonstrated for BMP2 (miR-106b, -20a, and miR-106a), Zinc finger and BTB domain containing 16 or ZBTB16 (miR-1271, miR-342), and Chemokine (C-X-C motif) ligand or CXCL3,6, and 8 (miR-106a/b, -20a, -493 and miR-425) via the HRMs indicated [49, 67, 68]. Of particular note, and in agreement with the scanty overlap in hypoxia-induced gene expression, we observed distinct patterns of association with pathways in cancer for hESC and hMSC virtually bisecting the associated target cartoon (S5 Fig). As indicated earlier this is highly suggestive of distinct HRM response and behavioural profiles in these two classes of stem cells; pluripotent and multipotent.

Overall, our findings add richly to the growing body of data surrounding hypoxic stem cell biology for both hESCs and hMSCs. In consideration of the strong influence of hypoxia on stem cell self-renewal and differentiation the regulatory and functional characterization of hypoxia regulated miRNAs or genes may provide strategies to design novel cellular therapies for regenerative medicine.

## Supporting Information

S1 Fig qRT-PCR data of **(a)** up-regulated and **(b)** down-regulated HRMs in SHEF2. QRT-PCR data showing levels of Drosha and DICER in hESCs grown in normoxia and hypoxia **(c)**. Graphical data points in a, b and c represent mean ± S.D. of a minimum of three independent experiments. (*P>0.01 and <0.05, **P<0.01).
(TIF)

S2 Fig Quantitative RT-PCR data of **(a)** up-regulated and **(b)** down-regulated HRMs in a different human bone-marrow derived MSCs. QRT-PCR data showing levels of Drosha and DICER in hMSCs grown in normoxia and hypoxia **(c)**. Graphical data points in a, b and c represent mean ± S.D. of a minimum of three independent experiments. (*P>0.01 and <0.05, **P<0.01).
(TIF)

S3 Fig qRT-PCR data showing inverse correlation of expression of (a) down-regulated and (b) up-regulated predicted target genes of HRMs in hESCs and similarly for hMSCs (c) downregulated and (d) upregulated. Graphical data points in a-d represent mean ± S.D. of a minimum of three independent experiments. (*P>0.01 and <0.05, **P<0.01).
(TIF)

S4 Fig Pathway analysis of hypoxia regulated miRNAs. A figure showing miRNA:target gene interaction network for HIF-1 signalling pathway in hESCs drawn using Cytoscape software. The green color refers up-regulation while the red color refers down-regulation.
(TIF)

S5 Fig Pathway analysis of hypoxia regulated miRNAs. A figure showing miRNA: target gene interaction network for pathways in cancer of hESCs **(a)** and hMSCs **(b)** drawn using Cytoscape software. The green color refers up-regulation while the red color refers down-regulation.
(TIF)

S1 Table List of upregulated (Sheet 1 and 3) and downregulated (Sheet 2 and 4) miRNAs showing >2 fold difference in response to hypoxia in SHEF1 cells and human bone marrow derived MSCs based on microarray expression profiling data. (XLSX)

S2 Table List of primers used for the detection of specific miRNAs using Quantitative RT-PCR. (DOCX)

S3 Table List showing location of HREs and their scores in the promoters of hypoxia induced miRNAs based on python program. (XLSX)

S4 Table List of predicted targets of up-regulated and down-regulated miRNAs in hE & MSCs using DIANA-MicroT-CDS program. (XLSX)

S5 Table List of analyzed pathways of hypoxia up-regulated (8a, b) and down-regulated (8c, d) miRNAs in hE & MSCs, respectively, using DIANA-mirPath program. (XLSX)

## Abbreviations

HIF1A: hypoxia inducible factor 1 A
HRM: hypoxia regulated miRNA
HREs: hypoxia response elements
hESCs: human embryonic stem cells
MSCs: mesenchymal stem cells
MAPK: mitogen activated protein kinase
NOG: Noggin
BMP: Bone morphogenetic protein
