# Supplementary figures and images for: Pluripotent and Multipotent Stem Cells Display Distinct Hypoxic miRNA Expression Profiles
Source: PLoS One. 2016 Oct 26;11(10):e0164976. doi: 10.1371/journal.pone.0164976 (PMC5081191; doi:10.1371/journal.pone.0164976)

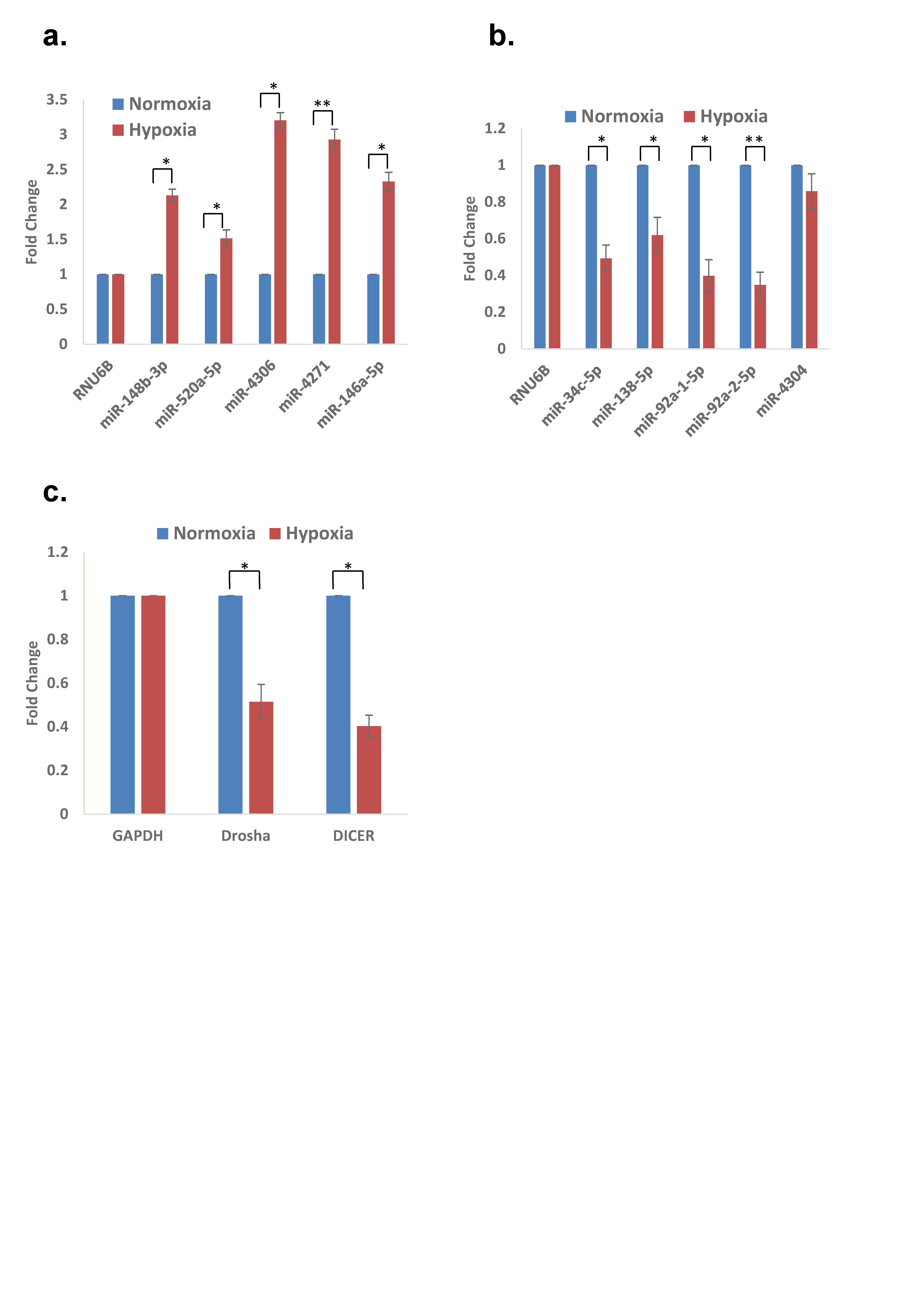

Supplement: S1 Fig — qRT-PCR data of (a) up-regulated and (b) down-regulated HRMs in SHEF2. QRT-PCR data showing levels of Drosha and DICER in hESCs grown in normoxia and hypoxia (c). Graphical data points in a, b and c represent mean ± S.D. of a minimum of three independent experiments. (*P>0.01 and <0.05, **P<0.01). (TIF) [file pone.0164976.s001.tif]

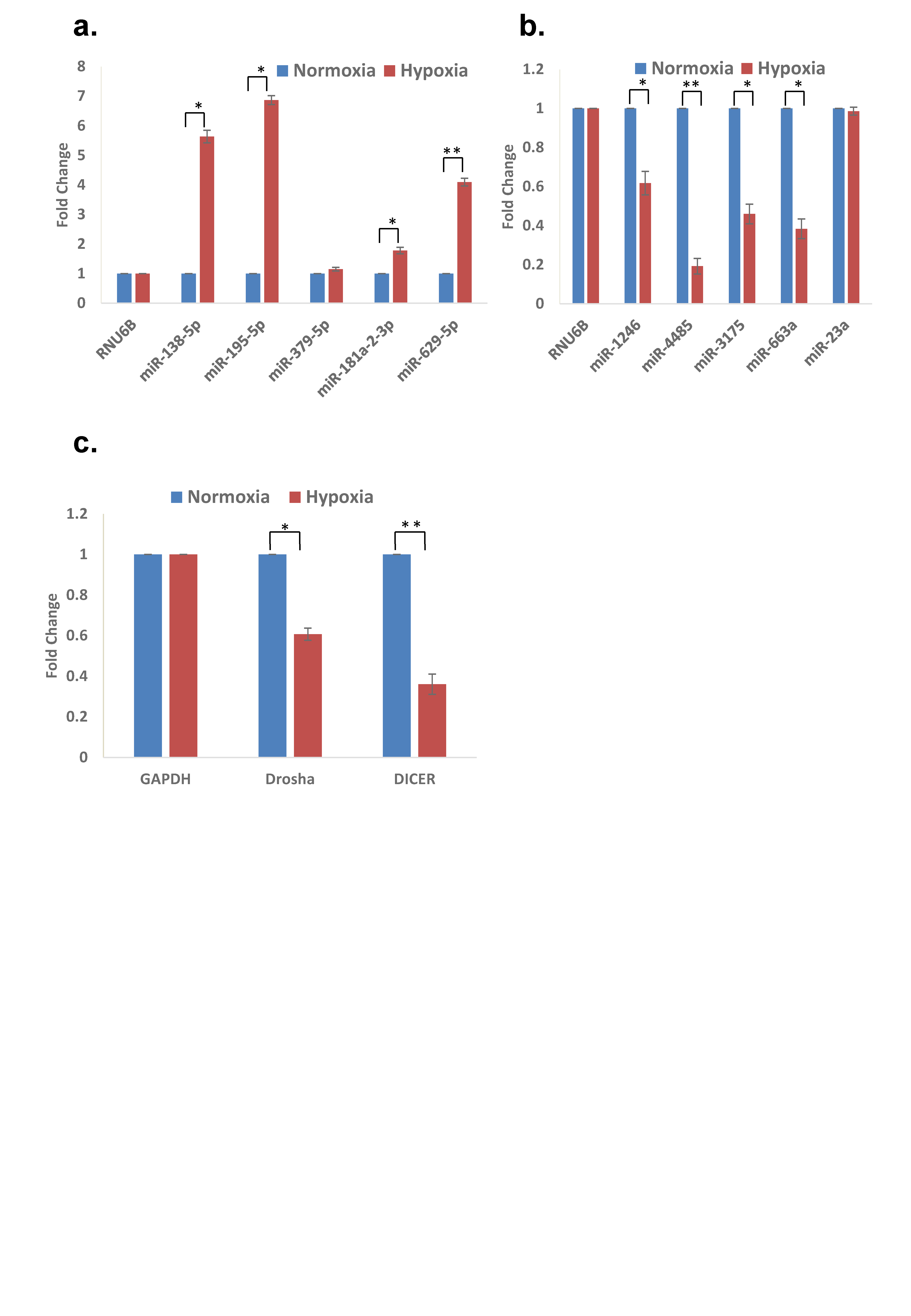

Supplement: S2 Fig — Quantitative RT-PCR data of (a) up-regulated and (b) down-regulated HRMs in a different human bone-marrow derived MSCs. QRT-PCR data showing levels of Drosha and DICER in hMSCs grown in normoxia and hypoxia (c). Graphical data points in a, b and c represent mean ± S.D. of a minimum of three independent experiments. (*P>0.01 and <0.05, **P<0.01). (TIF) [file pone.0164976.s002.tif]

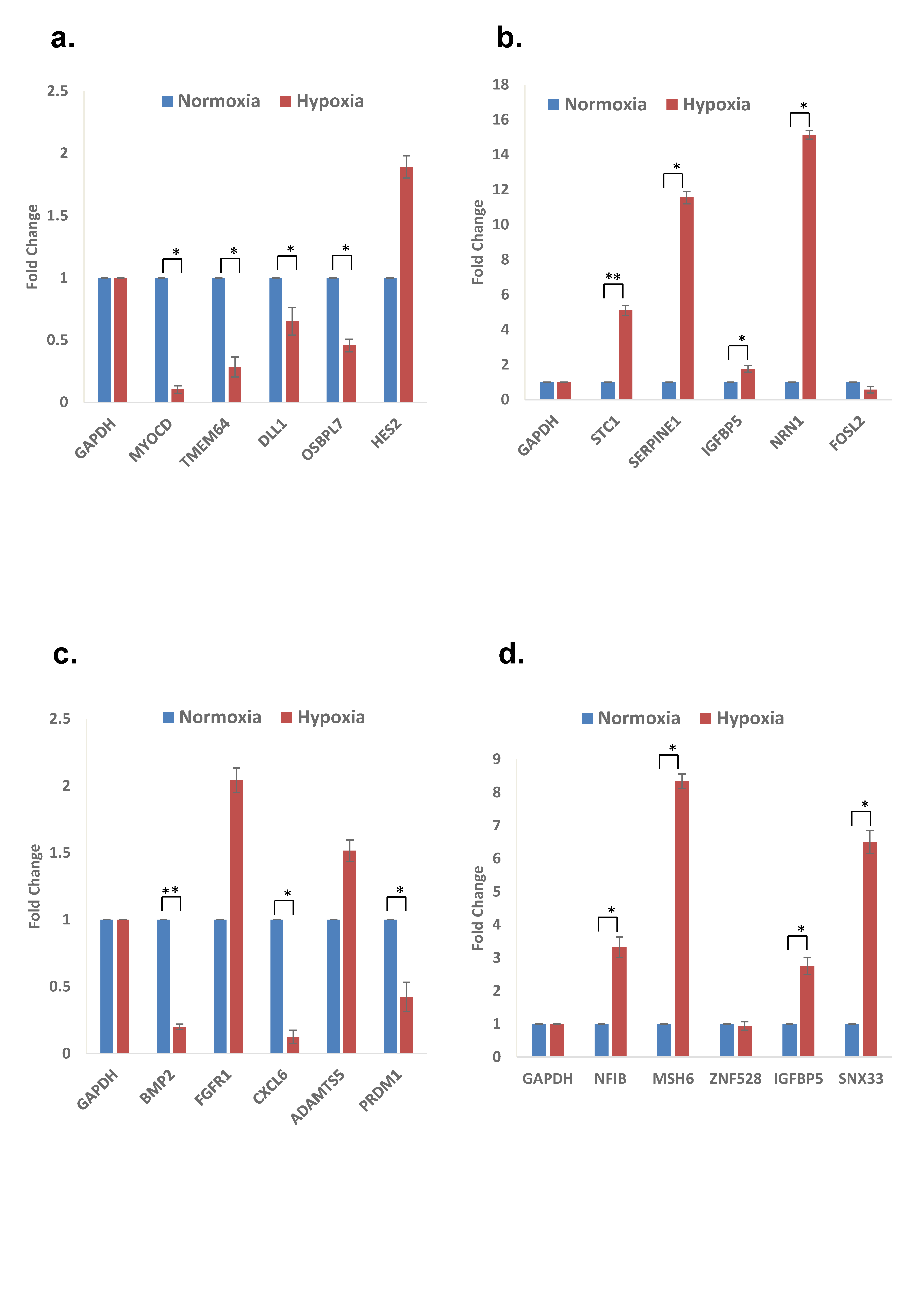

Supplement: S3 Fig — Graphical data points in a-d represent mean ± S.D. of a minimum of three independent experiments. (*P>0.01 and <0.05, **P<0.01). (TIF) [file pone.0164976.s003.tif]

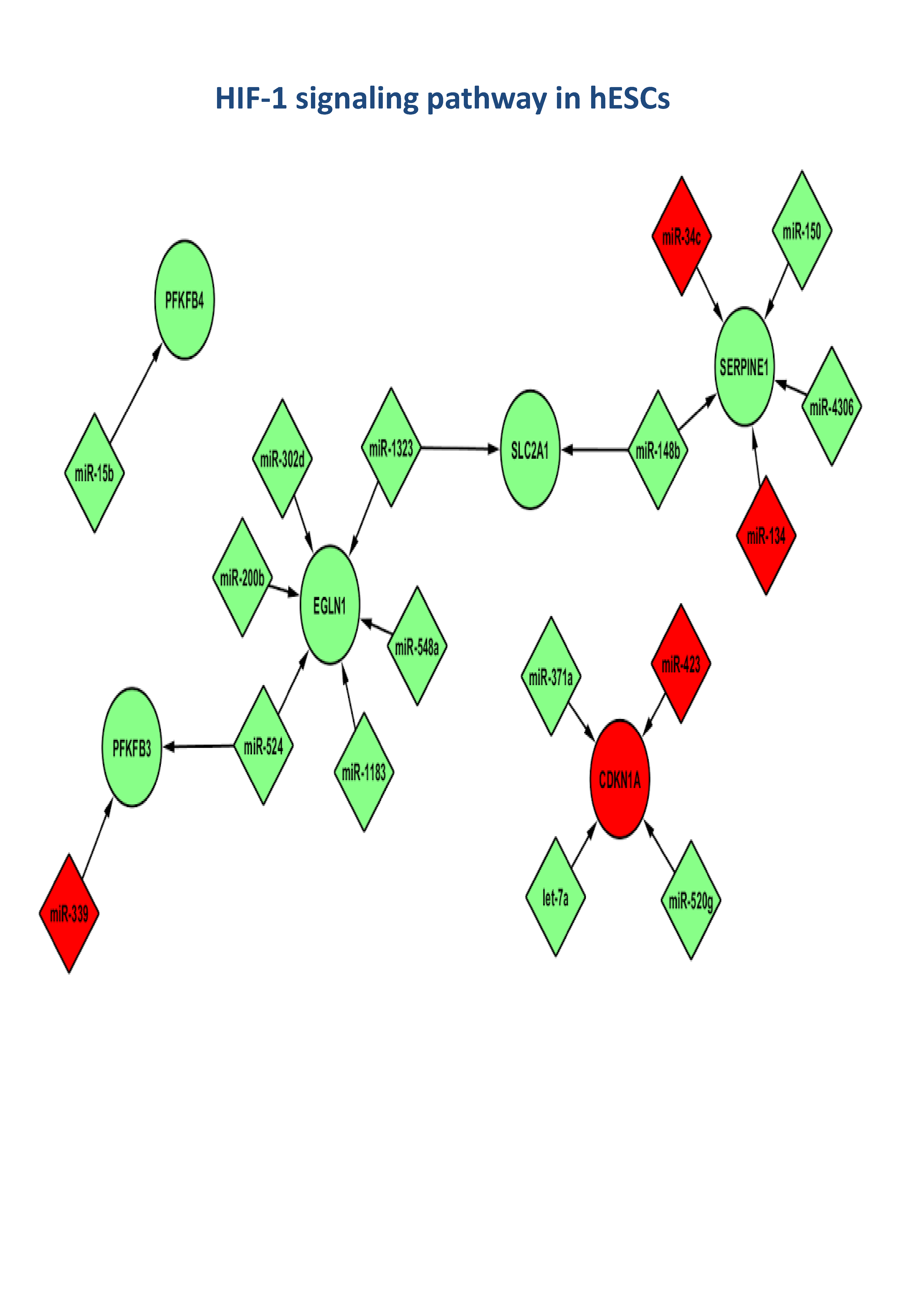

Supplement: S4 Fig — A figure showing miRNA:target gene interaction network for HIF-1 signalling pathway in hESCs drawn using Cytoscape software. The green color refers up-regulation while the red color refers down-regulation. (TIF) [file pone.0164976.s004.tif]

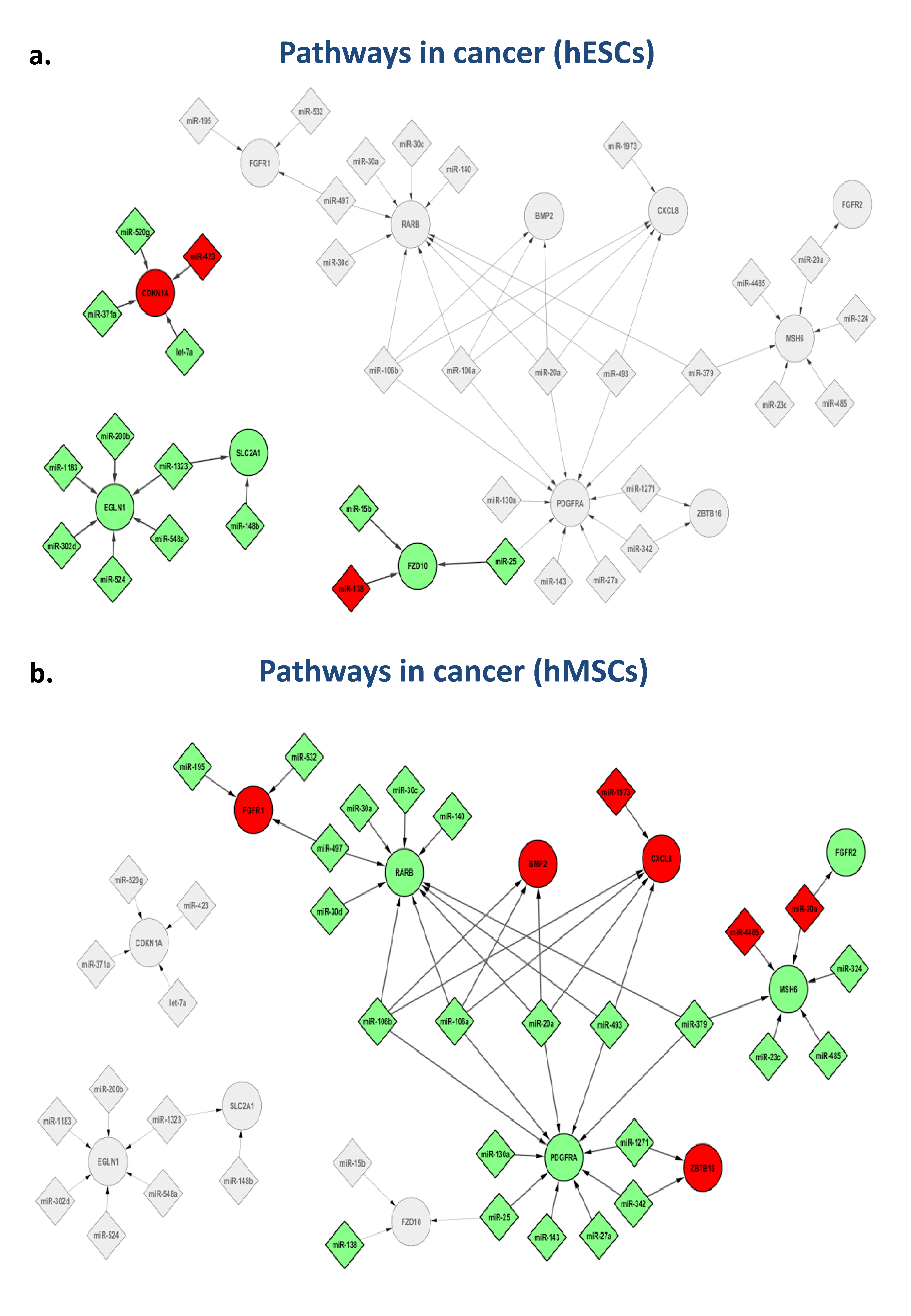

Supplement: S5 Fig — A figure showing miRNA: target gene interaction network for pathways in cancer of hESCs (a) and hMSCs (b) drawn using Cytoscape software. The green color refers up-regulation while the red color refers down-regulation. (TIF) [file pone.0164976.s005.tif]
