# Supplementary material for: Pluripotent and Multipotent Stem Cells Display Distinct Hypoxic miRNA Expression Profiles
Source: PLoS One. 2016 Oct 26;11(10):e0164976. doi: 10.1371/journal.pone.0164976 (PMC5081191; doi:10.1371/journal.pone.0164976)
Supplement: S2 Table — (DOCX) [file pone.0164976.s007.docx]

|  | **List of primers used** |
| --- | --- |
|  |  |
| **Primer detail** | **Primer sequence** |
| **Primers for detection of mature microRNA level** | |
|  |  |
| **MIR-4271 Forward Primer** | AGCTGGGGGAAGAAAAG |
| **MIR-4306 Forward Primer** | CTGCGCTGGAGAGAAAG |
| **MIR-520a-5p Forward Primer** | ATCGCTCCAGAGGGAAG |
| **MIR-148b-3p Forward Primer** | TACGCTCAGTGCATCACAG |
| **MIR-146a-5p Forward Primer** | GCGATGAGAACTGAATTCC |
| **MIR-92a-1-5p Forward Primer** | GAGGTTGGGATCGGTTG |
| **MIR-92a-2-5p Forward Primer** | AGGGTGGGGATTTGTTG |
| **MIR-34c-5p Forward Primer** | TGCAGGCAGTGTAGTTAGC |
| **MIR-4304 Forward Primer** | TAGCGTACCGGCATGTC |
| **MIR-138-5p Forward Primer** | GCGATAGCTGGTGTTGTG |
| **MIR-195-5p Forward Primer** | CGAGCGTAGCAGCACAG |
| **MIR-379-5p Forward Primer** | GCGCGTGGTAGACTATGG |
| **MIR-1246 Forward Primer** | GCGCGTAATGGATTTTTG |
| **MIR-4485 Forward Primer** | GTATATAACGGCCGCGG |
| **MIR-3175 Forward Primer** | ATATCGGGGAGAGAACGC |
| **MIR-663a Forward Primer** | ATATATAGGCGGGGCGC |
| **MIR-181a-2-3p Forward Primer** | GGACCACTGACCGTTGAC |
| **RNU6B**  **Forward Primer** | GCCCCTGCGCAAGGATGAC |
| **MIR-4271 RT Primer** | GTCGTATCCAGTGCAGGGTCCGAGGTATTCGCACTGGATACGACCCCCAC |
| **MIR-4306 RT Primer** | \|    \| GTCGTATCCAGTGCAGGGTCCGAGGTATTCGCACTGGATACGACTACTGC \| \| --- \| \| \| --- \| --- \| |
| **MIR-520a-5p RT Primer** | GTCGTATCCAGTGCAGGGTCCGAGGTATTCGCACTGGATACGACAGAAAG |
| **MIR-148b-3p RT Primer** | GTCGTATCCAGTGCAGGGTCCGAGGTATTCGCACTGGATACGACACAAAG |
| **MIR-146a-5p RT Primer** | GTCGTATCCAGTGCAGGGTCCGAGGTATTCGCACTGGATACGACAACCCA |
| **MIR-92a-1-5p RT Primer** | GTCGTATCCAGTGCAGGGTCCGAGGTATTCGCACTGGATACGACAGCATTG |
| **MIR-92a-2-5p RT Primer** | GTCGTATCCAGTGCAGGGTCCGAGGTATTCGCACTGGATACGACGTAATG |
| **MIR-34c-5p RT Primer** | GTCGTATCCAGTGCAGGGTCCGAGGTATTCGCACTGGATACGACGCAATC |
| **MIR-4304 RT Primer** | GTCGTATCCAGTGCAGGGTCCGAGGTATTCGCACTGGATACGACTGCCCTG |
| **MIR-138-5p RT Primer** | GTCGTATCCAGTGCAGGGTCCGAGGTATTCGCACTGGATACGACCGGCCTG |
| **MIR-195-5p RT Primer** | GTCGTATCCAGTGCAGGGTCCGAGGTATTCGCACTGGATACGACGCCAAT |
| **MIR-379-5p RT Primer** | GTCGTATCCAGTGCAGGGTCCGAGGTATTCGCACTGGATACGACCCTACG |
| **MIR-1246 RT Primer** | GTCGTATCCAGTGCAGGGTCCGAGGTATTCGCACTGGATACGACCCTGCTC |
| **MIR-4485 RT Primer** | GTCGTATCCAGTGCAGGGTCCGAGGTATTCGCACTGGATACGACTTAGGG |
| **MIR-3175 RT Primer** | GTCGTATCCAGTGCAGGGTCCGAGGTATTCGCACTGGATACGACACGTCAC |
| **MIR-663a RT Primer** | GTCGTATCCAGTGCAGGGTCCGAGGTATTCGCACTGGATACGACGCGGTC |
| **MIR-181a-2-3p RT Primer** | GTCGTATCCAGTGCAGGGTCCGAGGTATTCGCACTGGATACGACGGTACAG |
| **RNU6B RT**  **Primer** | GTCGTATCCAGTGCAGGGTCCGAGGTATTCGCACTGGATACGACAAAATATGGAAC |
| **Universal Reverse Primer** | GTGCAGGGTCCGAGGT |

|    \| \|  \| \| --- \| \| \| --- \| --- \| |
| --- | --- | --- |

|    \| \|    \|  \| \| --- \| \| \| --- \| --- \| \| \| --- \| --- \| --- \| |
| --- | --- | --- | --- |
